# Edible Bird’s Nest: The Functional Values of the Prized Animal-Based Bioproduct From Southeast Asia–A Review

**DOI:** 10.3389/fphar.2021.626233

**Published:** 2021-04-19

**Authors:** Ting Hun Lee, Waseem A. Wani, Chia Hau Lee, Kian Kai Cheng, Sheikh Shreaz, Syieluing Wong, Norfadilah Hamdan, Nurul Alia Azmi

**Affiliations:** ^1^ School of Chemical and Energy Engineering, Faculty of Engineering, Universiti Teknologi Malaysia, Johor, Malaysia; ^2^ Innovation Centre in Agritechnology for Advanced Bioprocessing, Universiti Teknologi Malaysia, Pagoh Research Center, Johor Darul Takzim, Malaysia; ^3^ Oral Microbiology General Facility Laboratory, Faculty of Dentistry, Health Sciences Center, Kuwait University, Safat, Kuwait

**Keywords:** edible bird's nest, bioproduct, functional values, anticancer, anti-aging, antioxidant

## Abstract

Edible Bird’s Nest (EBN) is the most prized health delicacy among the Chinese population in the world. Although some scientific characterization and its bioactivities have been studied and researched, no lights have been shed on its actual composition or mechanism. The aim of this review paper is to address the advances of EBN as a therapeutic animal bioproduct, challenges and future perspectives of research involving EBN. The methodology of this review primarily involved a thorough search from the literature undertaken on Web of Science (WoS) using the keyword “edible bird nest”. Other information were obtained from the field/market in Malaysia, one of the largest EBN-producing countries. This article collects and describes the publications related to EBN and its therapeutic with diverse functional values. EBN extracts display anti-aging effects, inhibition of influenza virus infection, alternative traditional medicine in athletes and cancer patients, corneal wound healing effects, stimulation of proliferation of human adipose-derived stem cells, potentiate of mitogenic response, epidermal growth factor-like activities, enhancement of bone strength and dermal thickness, eye care, neuroprotective and antioxidant effects. In-depth literature study based on scientific findings were carried out on EBN and its properties. More importantly, the future direction of EBN in research and development as health-promoting ingredients in food and the potential treatment of certain diseases have been outlined.

## Introduction

Edible Bird’s Nest (EBN) is a secretion created by swiftlets. *Erodramus* (echolocating swiftlets) and *Collocalia* (non-echolocating swiftlets) are among the two genera of swiftlets known to produce valuable EBN (Ma and Liu, 2012). Swiftlets are insectivorous birds, predominantly inhabited in South East Asia (SEA) and southern part of China (Aswir and Wan, 2010). The world’s largest producer of EBN is Indonesia, which has the largest colony of swiftlets currently, followed by Malaysia. (Hobbs, 2004). Saliva secreted from the pair of sublingual glands of swiftlets are the principal material used in the construction of the EBN. The sublingual glands of swiftlets increase in weight (2.5–160 mg) and reach their maximum secretory activity during nesting and breeding season (Jamalluddin et al., 2019). The male birds make nests by using their secretion to bind with some feathers and vegetation. The resulting material is shaped into nests with simultaneous attachment to the walls of the caves when is habituated in cave environment. In man-made premises, they are attached to the wooden linter (Lee et al., 2017). The nests are graded based on the dry mass, size, color, impurity and amount of feathers via physical appearance.

EBN has been the delicacy food in Traditional Chinese Medicine (TCM) since the Tang Dynasty (618–907 A.D.) (Marcone, 2005). EBN is cooked using double boiling method with rock sugar to make the Chinese cuisine, namely the bird’s nest soup (Hobbs, 2004). It was reported that Hong Kong is the largest importer of EBN globally, followed by the Chinese community from North America. EBN may be regarded as the most expensive animal by-product in the world, costing USD 2,000–10,000 per kilogram for its high nutritional and medicinal therapeutic values (Babji et al., 2015). The key component of EBN are glycoprotein, calcium, sodium, potassium and carbohydrate (Quek et al., 2018a). Owing to its esteem as a prized bioproduct in the East of the globe, EBN is also named as the “Caviar of the East” (Marcone, 2005). EBN has also been used as a health tonic in TCM due to its being a multipurpose general health rejuvenation tonic and social symbolic status delicacy during banquet (Ghassem et al., 2017). TCM claimed that EBN can treat malnutrition, improve metabolism rate, boost immune system and rejuvenate the skin complexion (Bashir et al., 2017). Moreover, in the modern research, EBN also exhibits some interesting therapeutic effects, such as anticancer, anti-aging, phlegm-dissolving, cough-suppressing, anti-tuberculosis, voice-improving, curing general debility and asthenia, and hastening recovery from illness and surgery (Daud et al., 2019a). There is a great amount of research taking place on the investigation of the hidden nutritive and pharmacological properties of EBN. Some of the reviews were focused on the authentication and identification methods of EBN, its bioactive components and food values (Lin et al., 2006; Wong, 2013; Lee et al., 2018). However, none of the reviewers have discussed the latest challenges facing by the researchers in EBN research field, such as the important substrates that contribute to the medicinal properties in EBN. Therefore, it is worthwhile to review the advances in the research involving EBN as a functional food from animal-based bioproducts and to discuss or address the challenges and future research perspectives of EBN. This manuscript will be served as a reference for the EBN researchers.

## Methodology

Published data from 2000 to 2019 were retrieved from the Web of Science (WoS), in August 2020, by using the following search string: TS = [(edible bird* nest*)]. Only publications in English language that report the functional values of EBN were included for subsequent analysis. The duplicate results were removed from the search. Current EBN trend observed in the Malaysia industry is also included in this review.

## The Trend of Edible Bird’s Nest Publications

**FIGURE 1:**
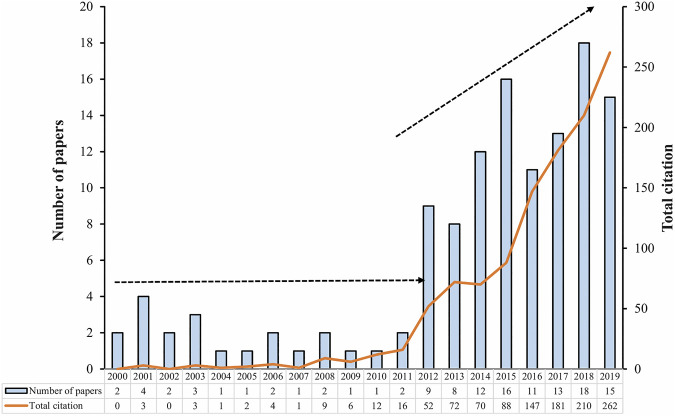
A pictorial depiction of the steadily growing interest in the research on EBN from 2000 to 2019.

A thorough search of the literature on WoS indicated that approximately 170 research publications consisting of various types of documents appeared in this topic. Out of 170 articles, only 124 publications were considered in this review which consists 119 original research articles and five review papers. The available publications discussed several aspects related to collection, extraction, purification, authentication, nutritive values, medicinal significance, and other important facts of EBN. According to the search and summarized in Figure 1, the publications on this topic remained low in 2000–2011. However, in 2012–2019, there is an increase in annual publications, with irregular trend. A noticeable and dramatic increase in publications numbers on this topic occurred since 2012. An increase on the number of citations has revealed the significant attention on the EBN work to the global scientific community.

## Overview of Edible Bird’s Nests

**FIGURE 2:**
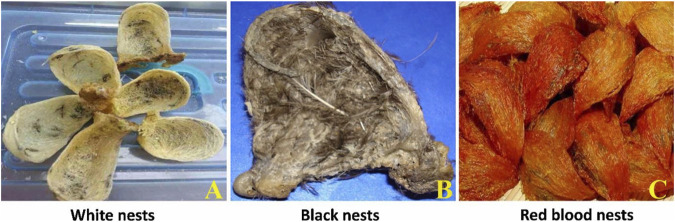
An overview of white **(A)**, black **(B)** and red **(C)** EBNs.

EBN is the hardened secretion produced by several species of swiftlets originally inhabiting in the limestone caves. EBN weighs at least 1–2 folds of the swiftlet’s body weight and can accommodate only the adult bird and nestlings. The swiftlets take around 35 days to complete the construction of the nest (Marcone, 2005). White nests (Figure 2A) are almost entirely made from saliva (Sims, 2008), while black nests (Figure 2B) comprised about 45–55% feathers and small dried leaves (Zulkifli et al., 2019). The white nests are mostly produced in the bird premises and only a little amount is found in the caves, whereas the black nests are only harvested in caves. Some slightly or entirely dull orange-red to brownish red nests called *Xueyan* or *Xueyanwo* in Chinese are occasionally found in caves and swiftlet houses. *Xueyan* is a Chinese word with the meaning “blood nest” or the blood-coloured nest which arise from the resemblance in the color of the blood. Red nests or blood nests (Figure 2C) are supposed to have higher health benefits and thus, fetch a higher price than white nests in the market (But et al., 2013). The EBN names deserved some special attention also. The first step is to identify them with color, for example, white nest, black nest and also red nest. White and black nests are explained above but the most interesting is the red or blood nests. Blood nest story was invented by the Hong Kong people where it is made to believe that the swiftlets will secrete blood (the best essence) when there is no more saliva to be used to build the nest. This makes the best quality nest (Lee et al., 2017). However, some researchers have suggested that red color might be due to the absorption of the minerals from the wall where the nest was attached (Wong et al., 2018a; Shim and Lee, 2018). Due to the higher price and hence better profit, some of the EBN processors decided to fake the blood nest with all kinds of dreadful methods. This has resulted in the “sodium nitrite crisis issues” that happened in 2011. China government has banned the import of EBN which caused multimillion dollar losses in Malaysia, after detecting a high content of sodium nitrite in some of the EBN. Subsequently, the Malaysian government has taken the initiative to standardize the EBN names based on the harvested location: the cave and house nest (Lee et al., 2017). It is categorized into only two major types based on the location where the nest is harvested. They totally did not acknowledge the red nest existence simply because there were too many ambiguous points to categorize them. After the ban was lifted in 2014, the content of sodium nitrite was controlled at 30 ppm which followed the Malaysian Food Regulation 1985 and Malaysia Standard MS 2334:2011 (Quek et al., 2015). Till today, Malaysia remains as one of the highest exporters of EBN to China.

## Traditional Value and Composition of Edible Bird’s Nest

EBN was once portrayed as a symbol of social status in ancient Chinese society (Jamalluddin et al., 2019) due to its rarity and high price. TCM prescribed EBN as the remedy for consumptive illnesses, *tuberculosis*, alleviating asthma, dry coughs, haemoptysis, asthenia, improving voice, difficulty in breathing, general weakness of bronchial ailment and relieving gastric troubles (Ghassem et al., 2017). Besides, EBN is traditionally believed to raise libido, fortify the immune system, promote growth, improve concentration, increase energy and metabolism, and regulate circulation (Bashir et al., 2017). Although the efficacy of EBN extracts in maintaining youthfulness and increasing physical strength have yet to be tested, but there is scientific evidence on EBN supplementation indicating that it could improve skin texture and alleviate the aging processes (Wong, 2013; Hwang et al., 2020). Based on these studies, EBN consumption may promote the human health.

**FIGURE 3:**
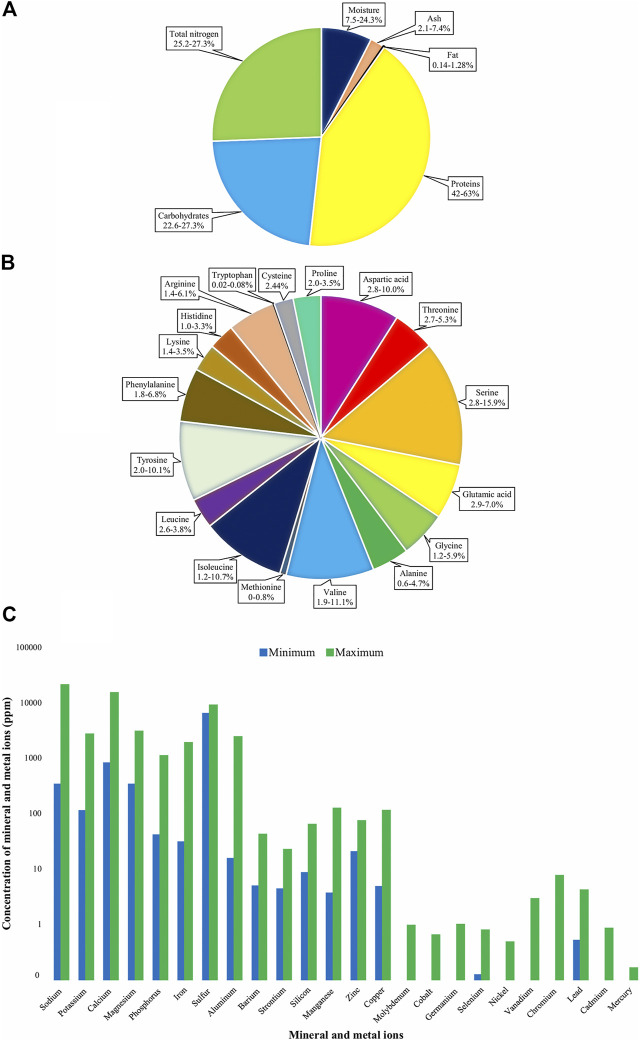
Total composition **(A)**, amino acid **(B)**, mineral and metal ions **(C)** in EBN (Marcone, 2005; Ma and Liu, 2012).

Protein is the major component in EBN which are commonly used for constructing the cells and tissues and consequently driving to other metabolic functions. Based on the previous studies, the average protein content in EBN is ranging from 50 to 55% of the dried weight (Wong et al., 2018c). In addition to the protein contents of EBN, carbohydrates form another major portion of its composition (Figure 3A) (Babji et al., 2018). The main carbohydrates present in EBN is sialic acid. Sialic acid facilitates development of gangliosides structure in the brain (Wang and Brand-Miller, 2003). Interestingly, ingestion of it can enhance and improve the neurological and intellectual for infants. Some other main and major ingredients in EBN are the essential trace elements such as calcium, phosphorus, iron, sodium, potassium, iodine and essential amino acids (Hun et al., 2015).

Based on these contents, EBN serves as a highly nutritious and health restorative food suitable for consumption by all age groups and genders. The modern analysis of its composition has been reported by many researchers as displayed in Figure 3. Out of the twenty types of amino acids desired by human, eighteen types of amino acids are detected in EBN. These include nine essential amino acids (phenylalanine, valine, threonine, histidine, tryptophan, isoleucine, methionine, lysine and leucine) required by human body for the growth and reparation of the tissue (Azmi et al., 2021). Out of nine essential amino acids, two of them, namely lysine and tryptophan, are not present in most plant protein. Hence, EBN could provide a complete amino acid for the vegetarians since it is categorized as vegan as it is not meat or animal blood.

Based on the content reported by various researchers, there were some differences in amino acid contents (Figure 3B). The actual causes of these differences are not known. However, these variance could be due to the EBN samples that were obtained from different places (Quek et al., 2018b). Also, the samples obtained could have been processed and adulterated (Huang et al., 2018). This is due to the fact that researchers could not standardize the EBN processing and cleaning method. Most of the time, samples were just obtained from sponsors or retailers but not knowing the actual process that had been carried out that make the variants.

The minerals and metal ions content (Figure 3C) in the EBN were either produced by the swiftlets (who built EBN) or leached from the environment. The content ranges are fairly wide as the samples were from various places and types. The excess mineral present in the food will cause negative effects and jeopardize human health, especially the heavy metal (Lead, Copper, Zinc, Mercury and Cadmium) when entering the human complex body through either inhalation, ingestion, and dermal contact. As described previously, some of these trace minerals and metal ions such as Lead, Mercury, Arsenic and Cadmium could have long term side effects in humans leading to various type of disease even at the small dose of ingestion or exposure (Zheng et al., 2020). Some of the heavy metal content in EBN showed in Figure 3C have alarming excess contents set by the majority food legislations (0–1 ppm). It is suggested that the heavy metal content limit should be enforced as this product is popular among children and more seriously, among pregnant ladies (Lee et al., 2017).

**FIGURE 4:**
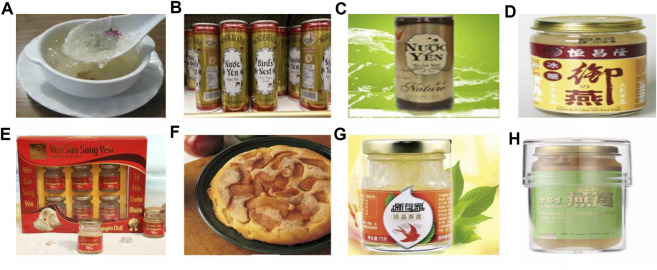
Some of the famous EBN market products based on foods labeled, outlook and, food products. **(A)** Bird’s nest soup, **(B)** Bird’s nest instant energy drink, **(C)** Vietnam bird’s nest powder, **(D)** Brand new concept EBN powder, **(E)** Bird’s nest drink, **(F)** Bird’s nest pudding recipe, **(G)** Instant Malaysian cubilose nourishing tonic and **(H)** Bird’s nest granules for supplements. The image is adapted from (Yen, 2015).

Traditionally, the benefit of EBN consumption in elderly include strengthening of lung and kidney, improving of the spleen, enhancing appetite and phlegm clearances. EBN helps to improve immunity in children, and strengthens the function of the kidney and lung in men (Quek et al., 2018a). Based on EBN’s content, in summary, EBN may be termed as a complete food enriched with a huge diversity of proteins, lipids, amino acids, carbohydrates, minerals and vitamins. Some of the essential amino acids, sialic acid, and other key constituents of EBN might have great health benefits in terms of general health especially on lung strengthening, improve skin health and anti-aging (Wang et al., 2019). Some of the recent developed EBN based products are shown in Figure 4. Till now, there has been little or none of the research on its functional and medicinal properties of EBN. It is further elaborated and discussed in the following section.

## Pre-Clinical Analysis and Therapeutic Effects of Edible Bird’s Nest

The effects of EBN extract have been summarized in Table 1 with details.

**TABLE 1:** Summary of studied effects using EBN extract.

Pharmacological activities	Sample preparation	Model	Dosage	Control group	Results	Proposed mechanism and suggested acting compound	References
Antiviral effects	Water extract (enzyme extraction)	Madin-darby canine kidney cells (MDCK)	4 mg/ml	Non-hydrolyzed EBN and untreated cells/mice	EBN after being hydrolyzed with pancreatin F, EBN showed potent antiviral properties in MDCK cells and prevented the virus’ hemagglutinin surface protein from binding to erythrocytes	The bioactive compounds (sialic acid or thymol derivatives) in EBN showed the potential effect toward the antiviral properties by inhibiting the viral genes (NA and NS1)	Haghani et al. (2016)
						Suggested acting compound: Sialic acid or thymol derivatives	
Anticancer effects	Water extract	Human colonic adenocarcinoma cell (Caco-2)	5 ppm	Untreated cells	Two commercial EBN samples showed 84 and 115% cell proliferation, respectively. The unprocessed EBN samples collected from 4 zones, east coast (91%), north (35%), and south (47%) also showed the potent in cell proliferation activity	It is suggested that some of the constituents of EBN must be imparting it with potential to kill rapidly dividing cancer cells	Aswir and Wan (2010)
	Acid extract	Macrophage cells (RAW)	5 ppm	Untreated cells	EBN decreases the production of anti-inflammatory TNF-α in RAW cells	The acting compound not suggested nor tested	
Human adipose-derived stem cells proliferation	Enzyme extraction	Human adipose-derived stem cells (hADSCs)	2000 ppm	Cells were cultures in control medium (DMEM +15% FBS)	EBN extract was strongly found to promote the proliferation of hADSCs mediated by the production of interleukin 6 (IL-6) and vascular endothelial growth factor (VEGF)	The production of IL-6 and VEGF was triggered by the activation of activator protein 1 (AP-1) and nuclear factor kappa-light-chain-enhancer of activated B cells (NF-κB). The EBN extract induced production of IL-6 and VEGF was inhibited by PD98059 [a p44/42 mitogen-activated protein kinase (MAPK) inhibitor], SB203580 (a p38 MAPK inhibitor) and ammonium pyrrolidinedithiocarbamate (PDTC; an NF-κB inhibitor). Thus, EBN extract-induced proliferation of hADSCs occurred primarily through amplified expression of IL-6 and VEGF genes, which was mediated by the activation of NF-κB and AP-1 through p44/42 MAPK and p38 MAPK.	Roh et al. (2012)
Epidermal growth factor-like activity	Water extract	Primary mouse embryonic fibroblast cell (3T3 fibroblasts)	0.016–2 ng/ml	Glucosamine	EBN stimulated thymidine incorporation in 3T3 fibroblasts cell culture, and this study proved the presence of EGF in EBN.	EGF are known to stimulate DNA synthesis. This study proved DNA synthesis occurred by detecting the present of thymidine	Dawson et al. (2005), Albishtue et al. (2018)
Enhancement of bone strength	Enzyme extraction	Female sprague-dawley rats	100 mg/kg	Fed an AIN93G-based normal diet	The femur of ovariectomized rats that treated with EBN extract showed the increment of calcium level and bone strength ability. Dermal thickness also increased and serum estradiol concentration was not affected by the administration of EBN extract	Estrogen production causes rapid bone loss during first decade after menopause. It was suggested that EBN extract effectively improve bone strength and at the same time regulates the serum estradiol concentration	Matsukawa et al. (2011), Chua et al. (2013), Hou et al. (2019)
	Hot-water extraction	Human articular chondroytes cell (HACs)	0.05–1.00%	Untreated cells	EBN extract increased HACs proliferation, reduced the catabolic genes expression and production of prostaglandin E2 (PGE2). In anabolic activity analysis, type II collagen, aggrecan and SOX-9 gene expression and total sulfated glycosaminoglycan production were increased	EBN extract able to down-regulate matrix metalloproteinases (MMP), cytokines and other catabolic mediator expression that can reduce destruction of cartilage and the degenerative progression of osteoarthritic cartilage	
Eye care effects	Water extract	Rabbit corneal keratocytes cell	0.05 and 1%	Untreated cells	Low concentration of EBN synergistically induced cell proliferation, especially in serum-containing medium. The corneal keratocytes reserved their phenotypes with the addition of EBN, which was confirmed by both phase contrast micrographs and gene expression analysis	EBN induce corneal cell proliferation and also capable to maintain their phenotypes and functionality by synthesizing stromal constituents in maintaining corneal cells	Abidin et al. (2011), Khalid et al. (2019)
						This confirmed by increased functional gene expression of collagen type 1, ALDH and lumican which are important corneal keratocytes cell proliferation	
						The acting compound not suggested nor tested	
Neuroprotective effects	Water extraction	Human neuroblastoma cell SH-SY5Y	100 μg/ml for crude extract and 200 μg/ml for water extract	Untreated cells	EBN treatment reduces the level of 6-hydroxydopamine-induced apoptotic changes in SH-SY5Y cells that was revealed by morphological and nuclear staining observations	EBN extract more potent in improving reactive oxygen species (ROS) build up, early apoptotic membrane phosphatidylserine externalization and the inhibition of caspase-3 cleavage. This report clearly indicated that EBN extracts might induce neuroprotective effects against 6-hydroxydopamine-induced degeneration of dopaminergic neurons via inhibition of apoptosis	Yew et al. (2014), Hou et al. (2015), Careena et al. (2018)
	Water extract	Human neuroblastoma cell (SH-SY5Y)	1,000 μg/ml	Untreated cells	EBN and its content (lactoferrin and ovotransferrin) attenuated H_2_O_2_- induced cytotoxicity and decreased radical oxygen species through increased scavenging activity	This report indicated that EBN acts as a neuroprotective (SH-SY5Y human neuroblastoma cell) agent against H_2_O_2_- induced cytotoxicity and cell oxidative stress	
						Suggested acting compound: Lactoferrin and ovotransferrin	
Antioxidant effects	Water extract	Human neuroblastoma cell (SH-SY5Y)	1,000 μg/ml	Untreated cells	EBN demonstrated protective effects against hydrogen peroxide-induced toxicity and cell oxidative stress on SH-SY5Y cells. Lactoferrin and ovotransferrin also possess antioxidant capacities on SH-SY5Y cells	EBN and its ingredients diminished hydrogen peroxide-induced cytotoxicity, and decreased ROS through increased scavenging activity. Lactoferrin and ovotransferrin in EBN could be contributing toward overall functional properties of EBN	Yida et al. (2014), Hou et al. (2015), Daud et al. (2019b)
						Suggested acting compound: Lactoferrin and ovotransferrin	
Erectile dysfunction	Water extract (enzyme extraction)	Castrated male wistar rats	1 mg/kg/day	Un-castrated rats	Castrated rat treated with 9 mg/kg/day of EBN extract exhibited significant higher testosterone and luteinizing hormone level. The penis index was observed to be significantly higher	Authors speculated with the increased dosage of EBN extract (9 mg/kg/day) that contributed to the sexual functions	Ma et al. (2012)
			3 and 9 mg/kg/day			Suggested acting compound: Testoterone but no evidence of EBN extract it contains	

Table 1 Summary of pre-clinical studies on the therapeutic effects of EBN extract.

### Antiviral Effects

Viruses are micro infectious agents which can only replicate in living cells of organisms that act as a host. Viruses can infect all living organisms that include plants, animals, bacteria and archaea (Koonin et al., 2006). Most of the viral infections have been reported as leading to lysis of the cell by changing the structure of cell membrane that result to the apoptosis of the host cells (Haghani et al., 2016). The most prevalence disorders due to viral infections are Influenza, Chickenpox, Cold Sores, Avian Influenza, Severe Acute Respiratory Syndrome (SARS) and Acquired Immunodeficiency Syndrome (AIDS).

Flu or influenza is a common viral infection that attacks the human population in the world. People who get infected by the influenza virus may be having varies symptoms like sore throat, muscle pains, running nose, headache, coughing, tired feeling and sometimes may come together with a high fever. Previous study on EBN has shown its potential to treat influenza virus infection in Madin-Darby Canine Kidney Epithelial (MDCK) cells. It also prevents human erythrocytes from undergoing hemagglutination by influenza A viruses (Haghani et al., 2016). Besides, after hydrolyzation with Pancreatin F, EBN extract also has reported the inhibition of the infection in a host range-depended manner with the human, porcine, and avian influenza viruses (Guo et al., 2006). The bioactive compounds such as sialic acid and thymol derivative have given EBN the potential to inhibit the virus. However, *Collocalia* mucoid or EBN contained a substrate for influenza virus sialidase Saengkrajang et al. (2013), whereby the inhibition can be disrupted by neuraminidase to some extent. Thus, EBN does not protect against influenza viruses sialidase. On the other hand, there was a report that suggested that the potential of EBN extract as an antivirus agent may be attributed to other inhibitory substances in the EBN that may be work together in a complex and bring the antiviral function in EBN. For instance, there was a previous study showed that N-acetylneuraminic acid may play a role in regulating the antiviral activity in EBN (Saengkrajang et al., 2013).

Interestingly, EBN displays no side effects to the MDCK cells and erythrocytes even at a high concentration of 4 mg/ml. Thus, EBN extract which has undergone Pancreatin F treatment and have a smaller molecular size is highly potential to be used in antiviral treatment due to its effectiveness and safety properties (Guo et al., 2006). Further study was carried out by Yagi et al. (2008) where they presented the N-glycosylation profile of EBN. The authors illustrated a tri-antennary N-glycan with the alpha 2,3-N-acetylneuraminic acid residues as a core component of the EBN. The authors further suggest that the sialylated high-antennary N-glycans are the core components that regulate the inhibition of influenza viral infection.

Overall, there are limited studies that demonstrated the antiviral properties of EBN. Thus, more analyses are needed to investigate the EBN antiviral activity toward the other pathogenic viruses. Although some of the antiviral activity in EBN have been attributed to the presence of N-acetylneuraminic acid and sialylated high-antennary N-glycans, it is important to ascertain other active ingredients in EBN that could possess antiviral properties. It is also vital to establish the active ingredients mechanisms of EBN that showed antiviral effects.

### Anticancer Effects

*Cancer* is one of the most common and lethal diseases after cardiovascular diseases (Zhao et al., 2016). It is a major public health havoc all over the world. Therefore, anticancer agents have always been of great interest (Ali et al., 2011; Saleem et al., 2013). Aswir and Wan (2010) documented the effects of EBN on the progression of epithelial colorectal adenocarcinoma cells (Caco-2 cells) in human. The EBN samples used two commercial brands and four unprocessed samples taken from the Department of Wildlife and National Parks, Kuala Lumpur. Analysis was done using 3-(4,5-dimethylthiazol-2-yl)-2,5-diphenyltetrazolium bromide (MTT) assay to determine the anticancer properties of the EBN samples. The authors observed that only 84 and 115% cells proliferated upon treatment using EBN samples from the two commercial brands. Nevertheless, the assay using unprocessed EBN samples from North, South and East Coast zones, resulted in 35, 47, and 91% of cell proliferation, respectively. It was reported the variations in the proliferation percentages of Caco-2 cells is subject to the type and source of EBN used (Aswir and Wan, 2010). These preliminary studies suggested that some of the constituents of EBN must be imparting into human body or cancer cells with potential to kill rapidly dividing cancer cells. However, the exact nature and the fate of the components of EBN responsible for the anticancer effects were not detected.

Complementary and Alternative Medicine (CAM) is a branch of medical and health care systems that include treatments and medications that are not regarded to be part of modern medical practices (Cassileth and Deng, 2004). CAM usage is quite popular among cancer patients. In Singapore, patients from western and eastern cultures who seek for cancer treatment were introduced to CAM practices either by taking TCM health supplements, traditional Indian medicine (Ayurvedic) or traditional Malay medicine (Jamu). Lim et al. (2006) documented EBN used in CAM for pediatric oncology patients in Singapore. The main therapeutic ingredients of CAM are alimentary changes, herbal supplement or tea and EBN. The authors suggested that CAM has a broad impact on every aspect of the healthcare system including pediatric oncology. In a similar fashion, Shih et al. (2009) documented the usage of EBN in CAM for adult cancer patients in Singapore. About 403 adult cancer patients who are taking medication at the Ambulatory Treatment Unit of National *Cancer* Center Singapore have answered a survey form. Based on the questionnaire analyses, 46% claimed taking EBN in their CAM and TCM. As part of the treatment. 54% of the respondents reported the use of EBN during CAM treatment to their oncologist and surprisingly, about 66.4% of the oncologists agreed with the application. Effectiveness from the combination of EBN and CAM to treat cancer is benefited by more than half of the patients. This report shows the advantages of EBN as alternative medicine in improving health of cancer patients.

The studies involving the anticancer evaluation of EBN extracts is yet to be carried out and tested on all range of cancer cells. Based on the literature found, most of the study were very preliminary and it is crucial for the EBN to be screened over a range of cancer cell lines so that a strong justification on its anticancer potential can be documented. Further investigations are needed to elucidate the exact role of different EBN constituents toward cancer cells.

### Human Adipose-Derived Stem Cells Proliferation

Stem cells are basic cells which are undifferentiated with a potential to differentiate into many different types of cells. Adipose Stem Cells (ASCs) are generally ubiquitous in all white adipose tissue. The pluripotent ASCs may find differentiation into other types of the mesenchymal cells, such as adipocytes, osteoblasts, chondrocytes and myocytes (Zuk et al., 2001; Zuk et al., 2002). Due to the mesodermal origin of adipose cells, their differentiation into neural tissue of ectodermal origin is unlikely (Tholpady et al., 2006). However, *in vitro* anti-oxidant activity of adipose cells revealed a bipolar morphology which is indistinguishable to neuronal cells (Boone et al., 2000). Stem cells are largely important in the regeneration or repair of aberrant or damaged tissues. ASCs are regarded as the most potent among the mesenchymal stem cells due to its ample confirmations of their pluripotency, multiplying capability and minimum donor morbidity (Ogawa, 2006). Besides, ASCs presence as highly potential agents in regenerative medicine as their cells can be collected in a huge volume with minimum donor-site morbidity. In the last decade, several studies have pointed to the use of ASCs in clinical applications in future. Roh et al. (2012) documented the induction of proliferation of Human Adipose-Derived Stem Cells (hADSCs) by EBN extract. EBN extract was revealed to stimulate the hADSCs cell proliferation *via* the production of vascular endothelial growth factor (VEGF) and Interleukin 6 (IL-6). The production of VEGF and IL-6 was triggered by the activation of nuclear factor kappa-light-chain-enhancer of activated B cells (NF-*κ*B) and activator protein 1 (AP-1). Interestingly, EBN extract also induced the production of VEGF and IL-6. The EBN extract induced production of VEGF and IL-6 was inhibited by PD98059 [a p44/42 mitogen-activated protein kinase (MAPK) inhibitor], SB203580 (a p38 MAPK inhibitor) and ammonium pyrrolidinedithiocarbamate (PDTC; an NF-*κ*B inhibitor), but not SP600125 [c-Jun N-terminal kinase (JNK) inhibitor]. Similarly, EBN extract-induced proliferation of hADSCs was also limited by PD98059, SB203580, and PDTC but not SP600125. Thus, EBN extract that promoted hADSCs proliferation mainly occurred by amplified IL-6 and VEGF genes that was mediated by the regulation of activation of NF-*κ*B and AP-1 through p44/42 MAPK and p38 MAPK.

In a nutshell, this report highlighted the potential of EBN extract for the improvement of the self-renewal of hADSCs through the enhancement of growth and multiply capability, which suggested that EBN extract may function as an external element to improve self-renewal by increasing the proliferative capacity of hADSCs. EBN extract affected the proliferation of typical healthy human cells only with no remarkable effects on modified cell lines or mutant cells, which indicated the cell specific effects of EBN extract toward normal cells. However, the details into which the active components of EBN were responsible for these effects still remain to be explored. Therefore, the details of specific component or a group of components of EBN that are responsible for these effects warrant further investigation.

### Epidermal Growth Factor-Like Activity

EGF promotes cell growth, dividing and proliferation by joining to the binding site at the epidermal growth factor receptor (EGFR). The size of Human EGF protein is 6,045 Da and it comprises three intramolecular disulfide bonds that linked together with 53 amino acid residues (Harris et al., 2003). EGF binds to the surface of EGFR with high affinity and activates the ligand-induced dimerization (Dawson et al., 2005). The binding will promote the activation of intrinsic protein-tyrosine kinase activity of the receptor. The activation of tyrosine kinase activity induces a signal transduction cascade in several biochemical changes within the cell such as the elevated of intracellular calcium levels, up regulated of the protein synthesis and glycolysis process, and increased of the expression level of the EGFR targeted gene. This leads to DNA synthesis and cell proliferation (Albishtue et al., 2018). Kong et al. (1987) was the first to demonstrate that there is a particular component in the EBN extract that has the EGF-like activity. The EGF-like substance was semi-purified from aqueous extract of the EBN using a Bio-Gel P-10. Following semi-purification, the EGF-like activity of EBN was identified using a series of biochemical analyses such as protein assays that include gel electrophoresis and competitive binding assays. Preliminary study using a specific radioreceptor assay showed the semi-purified EGF-like activity of EBN could generate a competitive binding curve that is parallel to the standard curve. Besides, the EGF-like component present in the EBN extract also could stimulate DNA synthesis by inducing the thymidine inclusion in the quiescent culture of the embryonic fibroblasts (3T3 fibroblasts). Analysis using heat treatment, trypsin digestion and mEGF (EGF isolated from mouse) antibody to investigate the simulation of DNA synthesis in human fibroblasts, the semi-purified EGF-like activity of EBN shown the ability to alter the stimulation of thymidine incorporation with the fibroblasts culture by restricted the trypsin digestion and eventually destroyed the its activity. Consistent results were recorded where the activity of mEGF and EGF-like activity derived from EBN were suppressed when treated with mEGF antibody. This result indicated that the nest EGF shared many similarities with EGF isolated from mouse or shrew in terms of its physical properties.

This section summarised the possible reason of EGF-like substance in the EBN that may contribute to its rejuvenating properties. However, there is a need to find out the possible substance, and characterize its structure through *in vitro* and *in vivo* studies, both alone and in EBN as a formulation.

### Enhancement of Bone Strength

Bones are rigid structures inside human body that form part of the skeleton system. They are vital to the support system and protect various important organs in the body. Additionally, bones play important roles in the production of white and red blood cells, minerals storage and involve in regulation of body movements and locomotion. Matsukawa et al. (2011) reported the increase of bone strength and dermal thickness of ovariectomized rats after daily consumption of EBN extract. It was reported that the oral consumption of EBN extract improved bone strength of ovariectomized rats due to the increasing of calcium level in the femur of the rats. More importantly, it was also observed the enhancement of dermal thickness following the EBN extract administration. Interestingly, EBN extract did not alter serum estradiol concentration level after EBN extract consumption. Since the ovarian production of estrogen will decline and this will be the major cause of rapid bone loss after menopause Gruber et al. (1984), Hou et al. (2019), EBN extract consumption can be an alternative and effective way to increase bone mass and at the same time slow skin aging in postmenopausal women.

Osteoarthritis (OA) is an established degenerative disorder caused by the deterioration of joints that includes the articular cartilage and subchondral bone. It is a painful disorder of the joints often causing stiffness and loss of ability. It is assumed that EBN extract poses some active ingredients that may minimized the occurrence of OA and contribute to the regeneration of cartilage (Wong et al., 2018b). In addition, Chua et al. (2013) documented the effects of EBN treatments toward the human articular chondrocytes (HACs) isolated from the knee joint of OA patients. They used hot-water extraction technique to obtain the EBN extract and reported that the supplementation of EBN extract results in increased HACs proliferation. In addition, EBN supplementation down-regulated the expression level of catabolic genes such as matrix metalloproteinases (MMP1 and MMP3), Interleukin 1, 6, and 8 (IL-1, IL-6, and IL-8), cyclooxygenase-2 (COX-2) and inducible nitric oxide synthase (iNOS) in cultured HACs. In addition, the production of PGE2 was significantly reduced in HACs. However, in anabolic activity analysis, total sulfated glycosaminoglycan production was increased and the expression level of targeted gens such as Aggrecan, type II collagen and SOX-9 were also elevated.

This research report clearly indicated that EBN extract has *in vitro* chondro-protection effect on HACs. However, it needs to be seen which components of EBN and how they demonstrate such effects. Therefore, EBN may be have a great potential as a replacement nutrient and supplement for osteoarthritis.

### Eye Care Effects

Eyes are the main sensory organs of the human body that react to light and give sight. The rod and cone cells in the retina are important conscious sense organs, which can enhance vision and conscious light perception. Our human eye is known to distinguish approximately 10 million colors (Land and Fernald, 1992). The transparent cornea covers three main organs which are pupil, iris, and the anterior chamber. Three distinct cell layers make up the cornea, including the stroma, epithelium and endothelium. Each individual cornea layer has its specialized visual functions. The cell layers also act as protective barriers from external environment (Lu et al., 2001). Approximately 90% of the corneal volume is comprised of the corneal stroma. It has a highly organized extracellular matrix (ECM) and consist of relatively low keratocyte density (West-Mays and Dwivedi, 2006). Keratocytes derived from the corneal layers are mesenchymal-derived cells which directly regulate in the synthesis and secretion of the ECM components (He and Bazan, 2008). Generally, the cornea is damaged by light injuries including localized burns, scraping or abrasions, and some extensive injuries in terms of surface or depth (Bizrah et al., 2019).

There are a few researches performed on the development of medicinal eye care product from EBN. Abidin et al. (2011) studied the effects of EBN on cultured animal corneal keratocytes through the *in vitro* study, including the isolation of corneal keratocytes with MTT assay in serum contained media (SCM) and serum free media (SFM), morphological observation for detection of phenotypes changes of keratocytes, and determination for gene expression of lumican, collagen type 1 and aldehyde dehydrogenase cells through Reverse Transcription Polymerase Chain Reaction (RT-PCR). Two significant results from Abidin et al. (2011) were reported for better recovery and tissue repair of eye. One significant result was the supplementary effect of EBN from 0.05 to 0.1% showed the highest cell proliferation and the capability to retain phenotypes of corneal keratocytes had been proved by the gene expression and phase-contrast micrographs (Abidin et al., 2011). Another study from Khalid et al. (2019) also clearly indicated that cell proliferation, especially in SCM, was synergistically induced by low EBN concentration. From these literatures, EBN showed great potential to enhance the cell repair from damage through higher cell proliferation rate and proper functioning maintenance in the wound healing of corneal tissues. To develop EBN-based eye drops products before *in vivo* application, the *in vitro* test can be a critical first step in the beginning. However, efforts are needed to see if there are any adverse reactions of EBN on other cell types in the vicinity of corneal keratocytes. Besides, it needs to be seen which of the EBN ingredients is responsible for the activity.

### Neuroprotective Effects

Neurodegeneration refers to the progressive loss of the structure or function of neurons. Several neurodegenerative diseases such as Parkinson’s (PD), Alzheimer’s (AD) and Huntington’s (HD), occur as eventual results of neurodegenerative processes. PD is an age-related progressive neurodegeneration. It is projected that the prevalence of PD will exceed nine million globally for people who aged more than 50 years old by the end of 2030 (Szatmari et al., 2019). Factors including depletion of dopaminergic neuronal in the substantia nigra and depletion of dopamine in the striatum are the hallmark pathology of PD (Yew et al., 2018). Przedborski (2005) revealed that the abnormal synthesis of α-synuclein (one protein type of presynaptic neuronal) had contributed to the neurodegenerative diseases. The degeneration of motor functions occurs with dopamine depletion and the patients often show clinical symptoms including slow responsiveness, rigidity, and tremor (Snyder and Adler, 2007).

For the past few years, works on EBN and neuroprotective effects have been studied by a number of scientist. For instance, the examination of neuroprotective effect on Human Neuroblastoma SH-SY5Y (HNS) cells using EBN extracts has been reported by Yew et al. (2014). The study showed that the pancreatin-digested EBN extract was inhibited the cell death of HNS cells up to 75 μg/ml while the maximum non-toxic dose was double (150 μg/ml) for EBN water extract. Nuclear staining and morphological observation indicated that the application of EBN can decrease apoptotic changes induced by 6-hydroxydopamine (6HD) in the HNS cells. Interestingly, cell viability significantly improved with digested EBN extract as compared to the EBN water extract. Nevertheless, EBN water extract showed great roles in the cleavage inhibition of caspase-3, regulate the early apoptotic effect on the phosphatidylserine externalization membrane and neuron recovery with reactive oxygen species build-up. Another similar study also clearly showed that enzyme extraction from EBN might possess neuroprotective effects through the apoptosis inhibition against 6HD-induced degeneration of dopaminergic neurons (Careena et al., 2018). EBN can therefore serve as a viable nutraceutical alternative for the protection against oxidative stress-related neurodegenerative diseases. A different study conducted by Hou et al. (2015) demonstrated the effect of EBN on the toxicity depletion of hydrogen peroxide (H_2_O_2_) on HNS cells. It was observed that lactoferrin and ovotransferrin within EBN attenuated (H_2_O_2_)-induced toxicity and cytotoxicity. The contents from EBN further decreased ROS with the enhancement of scavenging process which corresponds to a later work done by (Careena et al., 2018) where they found that EBN supplementation inhibited the production of oxidative markers ROS and TBARS in a Wister rat model of LPS-induced neuroinflammation. These reports indicated that EBN may act as a neuroprotective agent against cell oxidative stress and H_2_O_2_-induced cytotoxicity.

Although many researches have been reported on neuroprotective effects of EBN, the current scientific reports have not been able to demonstrate which of the specific EBN components or combination thereof has neuroprotective effects. Hence, the research efforts on EBN are needed to conclude this point in the near future.

### Antioxidant Effects

The human body has several anti-oxidant mechanisms that counteract oxidative stress from normal metabolic activity (Wang et al., 2019). The food contains the antioxidants components which are able to fight against the cell-disruptive effects. These antioxidants supplied function either individually or in combination with the endogenous systems. The implications of antioxidants with diet have been shown to be beneficial to human health, but their absence may trigger a number of diseases due to uncontrolled oxidative stress. Numerous vegetables and fruits have been shown to have antioxidant properties against certain cancers and other diseases. Thus, people who regularly rely on fruits and vegetables that are rich in anti-oxidants have lesser frequencies of free radical-induced diseases (Babji et al., 2018). Antioxidants have been the subject of great attention in the present scenario on because of their potential for fighting oxidative stress-related diseases.

EBN has long been first reported to contain antioxidants (Ghassem et al., 2017). As such, the effect of its antioxidants after oral administration is not fully known. EBN’s anti-oxidant properties are attributed to the pool of bioactive compounds such as amino acids, sialic acid, triacylglycerol, vitamins, lactoferrin, fatty acids, minerals, and glucosamine (Liu et al., 2012; Zainab et al., 2013; Lee et al., 2020). The anti-oxidative effect of EBN showed the presence of two main constituents, namely ovotransferrin and lactoferrin (Hou et al., 2015). The authors also reported their protective effects against H_2_O_2_-induced toxicity on HNS cells. Furthermore, transcriptional changes in anti-oxidant related genes were brought about by lactoferrin, ovotransferrin and EBN were in linked with the neuroprotection (Hou et al., 2015). Yida et al. (2014) documented the *in vitro* bio-accessibility and antioxidant properties of water extracts of EBN using Oxygen Radical Absorbance Capacity (ORAC) assays and 2,2-azinobis-(3-ethylbenzothiazoline-6-sulfonic acid) (ABTS) assay. It was observed that there were low antioxidant activity (about 1% at 1,000 μg/ml) on the undigested EBN water extract for both ABTS and ORAC assays. On the contrary, the digested EBN samples using pepsin, pancreatin and bile extract at similar concentrations showed improved antioxidant activities around 38 and 50% for ABTS and ORAC assays respectively. Besides, the EBN extracts showed non-toxicity toward human hepatocellular carcinoma (HEPG2) cells and protected HEPG2 cells from H_2_O_2_-induced toxicity. In short, enhancement of antioxidant activities of EBN after digestion highlighted some of its functional effects after consumption. However, further analysis such as *in vivo* studies are needed further characterize the significance of EBN from clinical perspective.

### Miscellaneous Effects

There are many therapeutic claims about EBN with no scientific proof which have been handed down from generation to generation. These include claims to treat *tuberculosis*, dry coughs, asthma, gastric trouble and stomach ulcer (Jamalluddin et al., 2019). Among the Chinese community, it is also renowned for its contribution to the fine porcelain complexion of Chinese beauties. In addition, it is also a normal practice to consume EBN among the Chinese mothers-to-be as a health supplement for both mother and child to have a pair of strong lung and fine complexion (Babji et al., 2018). These legendary claims on the EBN need further research so that the EBN can be developed into traditional medicine with scientific proofs, or at least, a scientifically credited functional food or health supplement. Erectile dysfunction is one of the main male sexual disorders characterized by tenacious inability to keep penile erection enough for pleasing sexual acts (Ma et al., 2012). This disorder mainly occurs due to a continuous spectrum of clinical factors such as difficulties, stress and physical illness in relationship (Corona and Maggi, 2010). Although drugs such as phosphodiesterase-5 inhibitors and testosterone supplication could help to overcome this disorder, the results of these treatments are not always desirable (Tsertsvadze et al., 2009). Ma et al. (2012) studied the effects of EBN on the sexual functioning of castrated rats and found that the seminal vesicle indices and prostate along with the hormone expression of endothelial nitric oxide synthase increased potently in the mouse groups that were treated with EBN. This result indicated that EBN has a potential to be an ideal active ingredient for the development of drugs in treating erectile dysfunction.

## Current Challenges and Future Perspectives

Despite the scarcity of science on the therapeutic effects of EBN in the past, several scientific papers have appeared on this subject especially for the past decades. Some of the research papers which have documented and summarized these effects include claim of antiviral, anticancer, proliferation effects on stem cells, epidermal growth factor-like activity, bone strength enhancement, eye care, neuroprotective antioxidant and other health-related effects of EBN. Research needs to be addressed so that the fundamental issues, including the molecular and biochemistry pathway of EBN to alleviate asthma, facilitate renal function, improve complexion, stamina and vitality bone health, could be fully understood. The specific components contributing to a specific function need to be identified. Besides, the correlations between dosages and activities of EBN need to be worked out also. Therefore, it would be a great breakthrough to discover the fundamental mechanisms by which the EBN component exerts both its biological effects *in vivo* and *in vitro* studies. Additionally, specific biological functions to specific components of EBN studies and then their isolation and purification component would be valuable. The conclusions and solutions would provide better use of EBN.

From the current literature updates, it can be concluded that EBNs collected from different sources and locations have their differences in composition. Therefore, it would be beneficial to standardize EBN composition and establish a standard operating procedure (SOP) to ensure that a stable and consistent outcome could be obtained. Further investigation focusing on the methodology reported including the complexity and variety of the location sources is needed to justify the variation that exists. If a sample is collected from the market, dealer or a retail shop, it is to be considered as processed since the probability of adulteration is high. One of the most common adulteration is bleaching so that the bird feathers cannot be seen. Others include addition of fortified materials to gain weight such as egg white, jelly, seaweed or even pork skin (Ma et al., 2019). These will definitely deviate the contents of EBN and hence affect the results of experiments.

EBN has long been used as a traditional remedy for some illness but has never been used as a medicine to cure or treat the sickness. This is simply due to the lack of study on the drug development and effective dose of this unique animal-based bioproduct. To the best of our knowledge, there is still no fractionation and isolation of single component work reported for EBN material leading to no specific component to demonstrate its therapeutic. There were only works done for *in vitro* and *in vivo* testing using the whole EBN extract with no further characterization on its single compound. Hence, due to insufficient scientific findings and reports, EBN could only regarded as food or at most remedy food.

The rise of allergic issues related to the consumption of EBN have been reported in several health cases. Allergic issues like skin rash, nasal obstruction and facial swelling have been reported in Japan after 5 min of consumption of EBN-contained dessert. The condition of allergic reactions can be in different degree of severity and some severe cases might cause death (Goh et al., 2000). One similar case was reported by National University of Singapore which documented that EBN caused food-induced anaphylaxis among the kids. The anaphylaxis occurrence might be due to the presence of putative allergens and abnormal regulation of Immunoglobulin E mediated process (Goh et al., 2000). Therefore, it becomes critical to identify whether a person is allergic or prone to allergy toward EBN protein by undergoing a skin prick test before consumption. These studies have set the precedence of EBN being an allergen. As the report comes from the highly reputable National University of Singapore, it is of great concern. However, as the test samples were obtained from the market or in other terms, it could have been adulterated by the bird’s premises handler or producer along the way in order to increase profit. The terms “egg white like” protein provides a good clue on this as the EBN processor normally will add egg white on the surface of EBN to provide the good looking luster on EBN to fetch a higher price (Guo et al., 2018). A better understanding and awareness of the consumer market norms and practices would ensure a good sample is applied for research to arrive at a more accurate conclusion.

There are issues arising in relation to the prescription of EBN to cancer patients. Generally, EGF receptors (which had been discussed in *Epidermal Growth Factor-Like Activity*) are highly expressed in several tumors cells such as non-small-cell lung, breast, colon, ovarian, renal, head and neck cancers (Herbst and Shin, 2002). Therefore, it may be assumed that EBN consumption might stimulate tumor progression and resist chemotherapy/radiation treatment in tumor cells. However, EBN also promotes healthy cell growth as explained earlier. *Cancer* patients should not avoid EBN as if it is a taboo merely based on EGF findings alone as EBN has been found to have apoptosis on cancer cells (Albishtue et al., 2018). Nevertheless, these concepts should be researched further to maximize the cancer prevention or treatments.

## Conclusion

The discussion in this article proves EBN could be a source of vital health-promoting ingredients with the reported content of amino acids, proteins, carbohydrates, fatty acids and minerals. The discovery of bioactivities in EBN are still in fetal stage and very much unexplored. Overall, the biological effects of EBN are still little explored as the available studies are very much preliminary and have been carried out on limited targets without any emphasis on *in vivo* studies. Thus, more exploration on the evaluation of bioactivities of EBN are needed to narrow the knowledge gap in EBN research studies. Like of the finding of subject or new material, the primary material should be standardized. The extract based on active ingredients or a standardized operating procedure should be filed. Despite some metabolite profiling studies, there are little information regarding the correlation of the specific active compound of EBN with specific medicinal effect. Furthermore, there is a lack of optimization studies related on the fractionation isolation and purification of active components that attributed to the bioactivities process in EBN. The present era demands further proteomic and genomic research to analyze EBN and its components for humanity’s welfare comprehensively. Finally, research should be encouraged to explore the biological and medicinal properties of EBN. There is a great need to study the correlations between the components and the functions of EBN so that some new and exciting compositions may be discovered. EBN, its extracts and products hold much for future development as possible food and medicinal-based products.
